# Green Preparation and Functional Properties of Reinforced All-Cellulose Membranes Made from Corn Straw

**DOI:** 10.3390/membranes14010016

**Published:** 2024-01-05

**Authors:** Wentao Zhang, Tianhao Wang, Zeming Jiang, Xin Gao, Changxia Sun, Liping Zhang

**Affiliations:** 1 College of Materials, Science and Technology, Beijing Forestry University, Beijing 100083, China; zhangwentao167@bjfu.edu.cn (W.Z.); 15002621992@163.com (T.W.);; 2 College of Science, Beijing Forestry University, Beijing 100083, China

**Keywords:** corn straw, regenerated cellulose membrane, all-cellulose nanocomposite, cellulose nanofibers

## Abstract

In this study, all-cellulose nanocomposite (ACNC) was successfully prepared through a green and sustainable approach by using corn stalk as raw material, water as regeneration solvent, and recyclable two-component ionic liquid/DMSO as the solvent to dissolve cellulose. The morphology and structural properties of ACNC were determined by scanning electron microscopy, transmission electron microscopy, Fourier transform infrared spectroscopy, and X-ray diffraction analysis, indicating homogeneity and good crystallinity. In addition, a comprehensive characterization of ACNC showed that CNF not only improved the thermal stability and mechanical characteristics of ACNC, but also significantly improved the oxygen barrier performance. The ACNC prepared in this work has a good appearance, smooth surface, and good optical transparency, which provides a potential application prospect for converting cellulose wastes such as corn straws into biodegradable packaging materials and electronic device encapsulation materials.

## 1. Introduction

Plastic film packaging materials, derived from petrochemical synthetic polymers such as polyethylene, are widely used because of their excellent properties, ease of processing, and low cost. At the same time, most of them are non-biodegradable and unsustainable, causing high consumption of petrol sources and having a huge impact on the environment. It is urgent to use natural renewable resources instead of petroleum-based plastics to prepare biodegradable packaging materials.

Cellulose is the most abundant resource in nature, and its films have biodegradability, excellent mechanical properties and sustainability, etc., and are used as potential materials for packaging and other applications [1]. However, cellulose is highly polar and hydrophilic, and interacts weakly with any non-polar or hydrophobic materials resulting in suboptimal properties of the final composite [2]. In the last decade, all-cellulose composite (ACC), as a kind of bio-composite made of only cellulose has emerged, which is expected to overcome the key problem of fiber-matrix adhesion in bio-composites by using chemically similar or identical cellulose materials as matrix and reinforcement materials, and is a potential green bio-composite packaging material [3].

There are two preparation methods for ACC. The first is a one-step method proposed by Gindl et al. [4], which achieves partial cellulose dissolution by controlling the dissolution conditions, and the dissolved part regenerates into the cellulose matrix in situ. The insoluble part remains as ACC reinforcement. However, the material properties depend largely on the degree of cellulose dissolution, which makes it difficult to achieve stable industrial production. The second preparation method includes two steps, in which insoluble cellulose is added to the fully dissolved part of the cellulose. In the process of solvent replacement, the dissolved cellulose is regenerated into a continuous matrix, while the undissolved cellulose acts as reinforcement in the composite material. This method was originally proposed by Nishino et al. [5]. Cellulose as a reinforcement phase includes regenerated cellulose fiber, cellulose microfilament, and nanocellulose. Among them, the ACC prepared by nanocellulose has a smooth surface and excellent optical transmittance due to its unique nano size effect. Therefore, it is of great significance to extract cellulose nanofiber (CNF) from cellulose material as a filler to prepare ACNC.

In addition to the inherent properties of reinforcement materials, many different combinations of material sources, substrates, and solvent systems also provide ACNC with a wide range of properties, and huge variations in formulations and processes also prove to exist [6]. Mature industrial applications depend on stable and limited yields of wood and cotton, and packaging materials are in high demand worldwide, so the development and utilization of cellulose from new sources is a particularly meaningful and important strategy [7]. Extensive research has been carried out on various sources of cellulose, mainly from natural wastes such as straw, ramie, sisal, durian, etc. Corn straw, as idle and huge reserves of resources, is one of the most intensively studied materials in the cellulose regeneration process, but its research in ACNC is limited. At the same time, the adaptability of solvents to different cellulose is different. For example, many solvents are only suitable for cellulose sources with a low average degree of polymerization (DP) and crystallinity [8]. Furthermore, the most commonly used solvent systems, such as LiCl/Dimethylacetamide (DMAc), NaOH/urea, and ionic liquid (IL), all have their own disadvantages. The disadvantage of LiCl/DMAc is that cellulose must be “activated” before it can be dissolved in the solvent, which is expensive and time-consuming [6]. NaOH/urea usually requires low temperatures typically below 4 °C [9], while most ILs require relatively high temperatures [10,11]. In addition, some solvent systems are also highly toxic, corrosive, and volatile, leading to a higher degree of health and safety-related issues [12]. It is of great significance to provide a green, stable, and controllable production method using a proper solvent system with strong adaptability to the properties of raw materials to prepare ACNC from agricultural waste.

In the preparation process of most ACNC, the actual content of ACNC is often difficult to determine because the solvent can dissolve both the reinforcing agent and the matrix, which greatly limits the stable industrial production potential of ACNC. In this study, corn straw with high DP was used as the raw material, and a two-component ionic liquid/dimethyl sulfoxide (DMSO) solvent system was used to dissolve the cellulose. We have achieved the quantitative addition of CNF to obtain ACNC by using the two-component IL/organic solvent system, which is a rarely explored process in terms of ACNC preparation [13]. It ensures that CNF, as the reinforcing agent, will not be eroded and fully dissolved, so as to control the actual content of CNF to further achieve excellent and desired performances. Meanwhile, both the IL and organic solvent DMSO are not involved in any of the reactions. Thus, they can be recycled and reused in practical industrial applications, which is both sustainable and economical. Finally, a quantitative and controllable method was provided for the preparation of ACNC with stable performance. The morphology and functional properties of the all-cellulose composites were systematically studied.

## 2. Materials and Methods

### 2.1. Materials

Corn straw pulp (CP) with 84% cellulose and 2.34% lignin content was purchased from Jilin Chemical Fiber (Jilin, China). The DP of corn pulp was determined to be 1700~1750 based on the solution of copper ethylenediamine hydroxide (CUEN) with Ubbelohde viscometer (Shanghai Qihang Glassware Factory, Shanghai, China), as described in [14]. All chemicals including DMSO, NaOH, ethanol, and ethylenediamine were of analytical grade and were used for preparation without further purification (Beijing Chemical Works, Beijing, China).

### 2.2. Preparation of CNF Suspensions in DMSO

**Figure 1:**
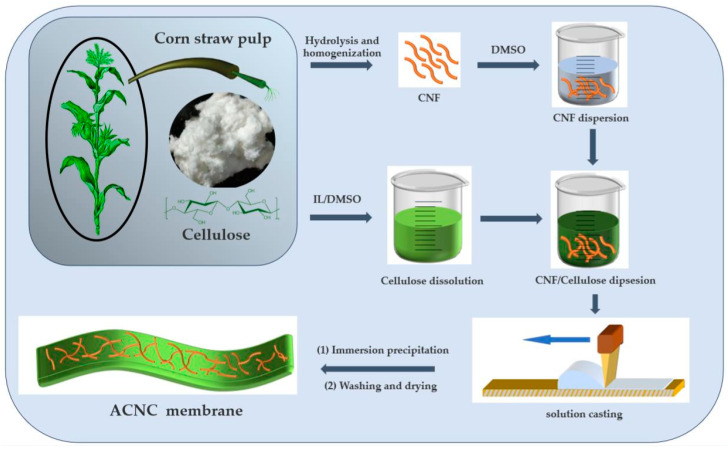
Schematic diagram of the ACNC preparation process.

The whole preparation process is represented schematically in Figure 1. The corn straw pulp board was added into 15.00 wt% sulfuric acid at 85 °C and stirred mechanically at 500 rpm for 4 h with a solid-liquid ratio of 1:40 (wt/wt). The suspension was washed with deionized water, centrifuged for concentration, and then dialyzed with water until it was neutral. The obtained water suspension was then centrifuged in DMSO dispersion for solvent exchange. The suspension was homogenized with ultra-Turrax (IKA) at 12,000 RPM for 15 min. Finally, the mixture was treated with high pressure viscosifier (NS1001S2K, GEA NiroSoavi Co., Cairate, Varese Province, Lombardy, Italy) for 10 passages at 790 MPa to obtain CNF suspension in DMSO. Different concentrations of CNF suspensions (0.15, 0.30, 0.60, and 0.90 wt%) were obtained by diluting this suspension.

### 2.3. Preparation of ACNC Membrane

The solvent system consisting of [Bu_4_N]^+^Ac^−^/DMSO was employed based on prior literature, where [Bu_4_N]^+^Ac^−^ refers to as tetrabutylammonium acetate [15]. As schematically illustrated in Figure 1, the two-component solvent was put into a 100-mL bottle, and 3.5 g of CP was added. The dissolution process was monitored with a polarizing microscope. At the temperature of 60 °C, most of the CP dissolved in 6 min, and the corn straw cellulose containing a small amount of lignin dissolved quickly in the two-component [Bu_4_N]^+^Ac^−^/DMSO solvent without pretreatment or activation, which was consistent with reference [16]. As shown in Figure S1, 0–60 s, the amorphous zone of cellulose was destroyed as the ionic liquid infiltrated the crystallization zone. At 360 s, dissolution was almost completed, and there were no crystal particles in the field of vision. Thus, a uniform cellulose solution was obtained by stirring at 300 rpm for 10 h at 60 °C in an oil bath (Table S1). 

The CNF dispersion and the cellulose solution were evenly mixed at 0–4 °C. Then, the liquid mixture was poured onto a clean glass plate, and the film thickness was adjusted to 300 μm with an automatic blade coater (Goldfull, Amoy, China). After blade coating, the film was left to stand for 30 s, and then was soaked in deionized water to remove the solvent system and obtain materials based on regenerated cellulose. The cellulose film was dried at 75 °C for 30 min using a drum dryer (No.2575-I, KRK, Kuki, Japan). The prepared regenerated cellulose membrane matrix was labeled as RC-C0, and ACNC samples with different CNF contents were labeled as RC-C2, RC-C4, RC-C8, and RC-C12 (CNF contents were 2.00 wt%, 4.00 wt%, 8.00 wt% and 12.00 wt% of cellulose in cellulose solution, respectively).

### 2.4. Characterization of CNF

#### 2.4.1. Determination of Mass Fraction

The constant dry weight of a clean weighing flask was recorded as *m*_0_. The CNF/DMSO suspension was weighed in the weighing flask, and the mass was recorded as *m*_1_. The flask and contents were then placed in a vacuum drying oven (120 °C, −0.1 MPa) for 6 h. After cooling to room temperature, the dried flask was weighed to constant weight. The process was repeated until the difference between the last two masses was less than 0.3 mg. The final dry mass was recorded as *m*_2_, and the mass fraction (*ws*) was calculated according to

(1) $$ws=\frac{m_{2}-m_{0}}{m_{1}-m_{0}}\times 100\%$$

#### 2.4.2. Transmission Electron Microscopy (TEM)

The morphology and size of CNF were determined by TEM (JEM-1010; JEOL, Tokyo, Japan) at 80 kV. CNF dispersion droplets were placed on a copper network containing carbon film coating to remove static electricity, and then negatively stained with phosphotungstic acid for analysis after drying.

### 2.5. Characterization of ACNC Membranes

#### 2.5.1. Fourier Transform Infrared (FTIR) Spectroscopy

The chemical composition of the ACNC films was investigated using FTIR spectroscopy (Vertex 70v; Bruker, Karlsruhe, Germany) with the ATR technique. The sample was vacuum-dried for 24 h before testing. First, the background air spectrum was collected with the ATR accessory before each sample measurement. Then, sample measurements were obtained by compressing the membrane sample onto the ATR crystal. All the spectral measurements were performed within the mid-infrared range of 4000–400 cm^−1^. Finally, the spectra were obtained with a resolution of 4 cm^−1^, accumulating 32 scans per spectrum.

#### 2.5.2. X-ray Diffraction (XRD) Spectroscopy

The aggregation structure of the ACNC films was analyzed by XRD spectroscopy using an XRD-6000 diffractometer (Shimadzu, Kyoto, Japan). Before measurement, the sample was vacuum dried for 24 h. Spectra were acquired using CuK alpha radiation (λ = 0.15418 nm) at 40 kV and 40 mA at a scan rate of 2°/min, with 2θ = 5° to 50°. Crystallinity was calculated using MDI Jade 5.0 software (Materials Data, Inc., Livermore, CA, USA).

#### 2.5.3. Scanning Electron Microscopy (SEM)

The ACNC film microstructure was characterized by SEM (S-3400N; Hitachi, Tokyo, Japan). The cellulose membranes were dried at room temperature and the mounted sample was sputter-coated with gold before observation using an SBC-12 sputter coater (Cressington, Watford, UK).

#### 2.5.4. Thermogravimetric Analysis (TGA)

The thermal properties of the membranes were characterized by TGA (Jupiter STA 449 F3; NETZSCH, Bavaria, Germany). Prior to analysis, the samples were dried for 3 h at 105 °C in a drying oven. Measurements were recorded under a nitrogen atmosphere with a heating rate of 10 °C/min from 50 °C to 450 °C.

#### 2.5.5. Mechanical Properties

The membrane sample was cut into portions measuring 10 × 60 mm. The sample was air-dried at room temperature for 24 h and the thickness of each sample was measured with a micrometer. The tensile strength and elongation at breakage of the dry samples were measured by fixing the two ends of the samples on the electronic tensile apparatus. The pulling speed of the moving head was 5 mm/min, and 15 portions from each sample were tested and the data averaged.

#### 2.5.6. Oxygen Permeability Measurements

Oxygen transmission rates of RC-C0 and the ACNC films were determined at 23 °C and 50% and 80% RH (Relative Humidity) using a Labthink C203H (Labthink., Jinan, Shandong Province, China) under standard conditions (ASTM 3985). Each measurement was continued until the O_2_ transmission rate reached a stable value. The oxygen permeability was determined from the oxygen transmission rate and the film thickness. Each sample was divided into three 10 × 10 cm^2^ subsamples and each subsample was measured. The results were obtained by calculating their average values. The relative standard deviations for each film were within 5%. 

#### 2.5.7. Transparency Measurements

The optical transmittance of the film was measured by Shimadzu UV-1800 (Shimadzu, Kyoto, Japan) spectrophotometer in the range of 380–780 nm. The sample of each regenerated cellulose film was placed directly on the sample holder, without using a colorimeter, and the data were normalized according to the thickness.

## 3. Results and Discussion

### 3.1. Morphology Analysis of ACNC

**Figure 2:**
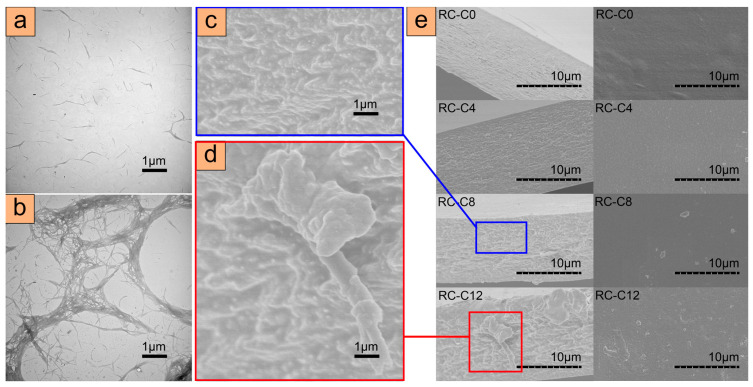
(**a**,**b**) TEM images of CNF; (**c**,**d**) Enlarged SEM images of RC-C8 and RC-C12. (**e**) Cross-section (**left**) and top-view (**right**) SEM images of ACNC with different CNF contents.

The micro morphologies of corn straw CNF isolated by acid hydrolysis processes and decomposed under strong mechanical action were observed in Figure 2a,b. The fibers showed slight aggregation, probably because of hydrogen bonding. As seen in Figure 2a,b, the CNF showed a rod-like or filamentary structure with a width of about 30–60 nm and a length of 350–1500 nm. These results were similar to those reported in previous studies, which suggested that the CNFs were successfully prepared [17,18]. 

It is well known that good dispersion of the reinforcement phase in the matrix is the key factor to improve the properties of composites. In order to explore the dispersion of CNF in the matrix, SEM analysis was carried out on the cross section (left) and top view (right) of ACNC with different CNF contents (Figure 2e). 

From the top-view SEM images, the RC-C0 in the absence of CNFs showed surface relief, while RC-C4 and RC-C8 showed small aggregated particles, and all three samples displayed a moderate surface roughness. However, it is not possible to observe the individual fibers in the films due to their small size. From the cross-section SEM images, the RC-C0 and RC-C4 were more homogeneous than RC-C8. The distribution of CNFs in the composite films containing 12% CNFs was not as ideal as their distribution in films containing 8% or fewer CNFs. This may be due to the selective agglomeration of CNFs which led to the inhomogeneity of the CNFs, as suggested by similar results in prior literature [19].

For pure regenerated cellulose membrane RC-C0, there were many nano-scale defects, because it is difficult to control the uniformity of solvent diffusion through rapid solvent exchange when cellulose solution is in contact with a coagulation bath [20]. The defects, resulting in stress concentration of the film and insufficient cellulose adhesion, may have a bad influence on the mechanical performance [21]. According to the SEM images in Figure 2e, in the samples with the moderate addition of CNF (RC-C4 and RC-C8), the reinforcing agent CNF displayed a uniform and orderly distribution in the cellulose matrix. The morphology indicated excellent compatibility between the reinforcing agent and the matrix, which was also beneficial to a good mechanical performance. The stress on the substrate can easily be transferred to the reinforcing material, thus preventing the growth of cracks [20]. However, as observed at the RC-C12 interface, locally high bulk solids were formed under a higher cellulose phase load, while surrounding voids were observed due to the lack of a matrix for the cementitious phase [13]. Compared with ACNC prepared by similar methods and different solvent systems in other studies on corn stalk cellulose, the cross section of CNF is still compact and has almost no defects when the CNF content is 8 wt%, which is of great significance for further improving mechanical properties [22].

### 3.2. Crystallinity, Molecular Structure, and Thermal Stability Analysis

**Figure 3:**
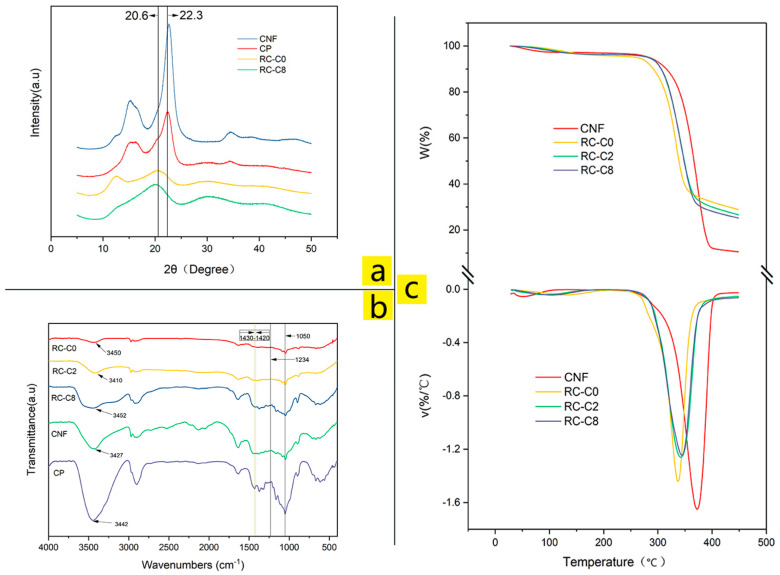
(**a**) X-ray diffractograms of (top to bottom) CNF, CP, RC-C0, and RC-C8. (**b**) FTIR spectra of (top to bottom) RC-C0, RC-C2, RC-C8, CNF, and CP. (**c**) TGA and DTG scans of (top to bottom) CNF, RC-C0, RC-C2, and RC-C8.

The X-ray diffraction spectra of CP, CNF, RC-C0, and RC-C8 are shown in Figure 3a. Left peaks at 14.9°, 16.6°, 22.6°, and 34.5°, which were present in the typical cellulose I crystal diffraction pattern, correspond to the crystal planes of (1$\bar{1}$0), (110), (200), and (004) and are visible in the diffraction patterns of straw fiber pulp and CNF [23,24]. The results indicate that after sulfuric acid hydrolysis and mechanical homogenization, the crystal morphology of the cellulose did not change, and CNF still presented the crystal morphology of cellulose I. Pure regenerated cellulose membrane (RC-C0) showed peaks at 12.5°, 20.6°, and 22.3° that corresponded to the (1$\bar{1}$0), (110), and (200) crystal planes [25,26], and suggested that regenerated cellulose showed some conversion to the type II crystal structure. The peak shape of the prepared ACNC (RC-C8) was more similar to that of the regenerated cellulose (RC-C0), which proved that cellulose II had an advantage in ACNC. Comparison between RC-C0 and RC-C8 showed that RC-C8 had more obvious shoulder peaks at around 12.5°, 14.9°, and 16.6°. At the same time, the peak of RC-C8 at 20.6° was sharper than that of RC-C0, because the diffraction peak of 22.6° in CNF was superposed with it [15]. This indicated that cellulose I and cellulose II coexisted in ACNC, suggesting that CNF was successfully introduced into the cellulose matrix. The crystal types of cellulose include type I, II, III, and IV, with type I having the best mechanical properties. Therefore, it is expected that the composites will have better tensile properties [27]. According to MDI Jade5.0, the crystallinities of CP, CNF, RC-C0, and RC-C8 were 61%, 79%, 37%, and 51%, respectively. It is concluded that the presence of CNF leads to improved crystallinity of the regenerated cellulose membranes.

Figure 3b illustrates the FTIR spectra of samples of CP, CNF, pure regenerated cellulose membrane, and ACNC membranes used to characterize isomers and mixed crystals. The spectra all show similar contours which indicated that no additional chemical reactions occurred, and no derivatives were generated during the preparation of the regenerated cellulose membrane. The peaks at 1234 cm^−1^ in all of the spectra corresponded to the syringic ring and C–O stretching of lignin and xylan [28], indicating that a small part of lignin existed in the raw material and the samples. The strong absorption peak at 1050 cm^−1^ belongs to the frame vibration of the cellulose (C–O–C) glycosidic bond [26], while the absorption band at around 1420–1430 cm^−1^ is related to the CH_2_ scissoring motion at C6 and linked with the portion of crystalline structure in cellulose. The band that occurred at 1420 cm^−1^ is characteristic of cellulose II and amorphous cellulose. This band may be shifted to 1430 cm^−1^ when the content of cellulose I was higher [29], just as the CNF and CP showed. With the increased content of CNF in ACNC, the absorption band at 1430 cm^−1^ was stronger, which pointed to the ACNC membrane as a typical mixed crystalline cellulose material in which the crystal structure of CNF was well preserved. The wide peaks at around 3400 cm^−1^ can be attributed to stretching vibration of –OH [30]. 

TGA measurements were performed to investigate the thermal stability of CNF, RC-C0, and ACNC. TGA and DTG curves are shown in Figure 3c. At about 100 °C, free water and bound water in the material were lost [17]. It is found that the main stage of mass loss of ACNC appeared in the range of 250 °C to 380 °C. At this stage, all samples began to decompose with volatilization similar to the cellulose-based materials described in [31]. Determined by the DTG curves, the maximum decomposition temperature (T_dmax_) of RC-C0, RC-C2, RC-C8, and CNF were 339.0, 342.1, 343.6, and 372.6 °C, respectively, and the T_dmax_ of CNF was significantly higher than that of RC-CO. This can be attributed to the differences in the crystallinity of cellulose films and the different interfacial interactions between cellulose polymers and cellulose nanofibers [23]. ACNC fell somewhere between RC-C0 and CNF, and the T_dmax_ increased with the increase of CNF content, revealing the positive effect of CNF on the thermal stability of composites. The improvement of gas barrier properties also makes it difficult for decomposition gas to be released from the nanocomposite, which also delays the degradation of the film to a certain extent [32]. The higher thermal stability of the fillers with higher crystallinity index may also be the reason for the improvement of the thermal stability of the composites studied [33].

### 3.3. Functional Properties of All-Biomass Films

**Figure 4:**
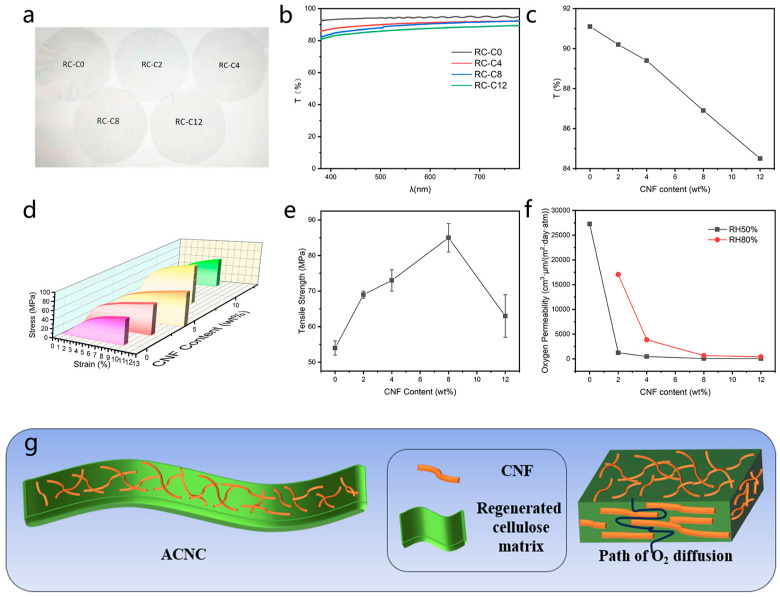
(**a**) Photos of ACNC. (**b**) Optical transmittance of ACNC with different CNF contents in visible light range. (**c**) Optical transmittance of ACNC with different CNF contents at 550 nm. (**d**) Stress–strain curves of ACNC films with different CNF contents. (**e**) Tensile strength of ACNC with different CNF contents. (**f**) Oxygen permeability of ACNC with different CNF contents at RH50% and RH80%. (**g**) Schematic oxygen diffusion through ACNC.

The digital photo of ACNC is shown in Figure 4a. By naked eye observation, the film surface is smooth and uniform. The text of the two images can be seen very clearly through the film. The high transparency of ACNC can be attributed to efficient interfacial contact between CNF and the matrix and the moderate surface roughness of RCNC [8,34]. The transmittance results in the range 380–780 nm and at 550 nm are shown in Figure 4b and Figure 4c, respectively. The transmittance of all composite membrane materials at 550 nm was almost higher than 85%, and the introduction of CNF did not significantly weaken the high transparency of cellulose membranes. Specifically, the optical transmittance values of RC-C2, RC-C4, RC-C8, and RC-C12 at 550 nm were 90.2%, 89.4%, 86.9%, and 84.5%, respectively. The good transparency of ACNC membrane is also attributed to the better distribution of CNF in the cellulose matrix shown in SEM images. It can be observed from SEM images in Figure 2e that in RC-C4 and RC-C8, CNF was uniformly distributed in the matrix, which is beneficial to a high transparency and also consistent with the optical transmittance results. The similar results were observed in prior literature that the degree of transparency of the all-cellulose nanocomposite films reflects the status of dispersion of CNF in the cellulose matrix. The reason may be that inhomogeneous distribution of CNF causes large size agglomerates, resulting in scattering and thus the decrease in optical transmittance [23]. At the same time, with the increase of CNF content, the optical performance of ACNC decreased continuously, because the transparency of all-cellulose nanocomposite film depends on the size effect and dispersion state of cellulose nanofibers. But ACNC still has better transparency than several other cellulose-based nanocomposites in visible light range, such as cellulose-graphene (below 70%), graphene oxide (below 80%), and carbon nanotube (typically below 25%) nanocomposites. The comparison between our results and literature data is summarized in Table S2. Therefore, these straw-based, optically good all-biomass films were manufactured through a new, green, and stable process that can be tried for some optical devices.

The mechanical properties of ACNC with different CNF contents are characterized by elongation at break and tensile strength. Compared with the traditional composite materials used today, the advantage of ACNC is the almost perfect chemical bonding at the interface between the matrixes. The matrix and reinforcement materials are chemically identical in favor of effective stress transfer and adhesion at their interfaces [35]. As shown in Figure 4d,e, with the addition of CNF, the mechanical properties of ACNC were significantly improved. Notably, the elongations at break of RC-C4 was as high as 13%. The tensile strength of all CNF-reinforced ACNC samples were higher than that of RC-C0, with RC-C8 exhibiting the highest value of 87 MPa. These results are summarized in Table S3. The significant improvement in mechanical properties may be attributed to the strong interfacial interaction between the matrix and reinforcement phase, which transferred stress from the matrix to CNF, a rigid fiber with excellent mechanical properties [36]. Although both the matrix and the enhancement phase were composed of cellulose, the enhancement efficiency of the cellulose enhancement phase showed improvement only at low concentrations. When the concentration of the cellulose reinforcement phase in the matrix was too high, CNF aggregation occurred, which may greatly limit the improvement of the mechanical properties of ACNC [37,38]. Combined with the SEM (Figure 2d) analysis, it may be that some of the CNF agglomerated with high orientation, which resulted in the mechanical weakness perpendicular to this orientation. In addition, the composite film prepared from corn straw cellulose obtained in this study has a similar and relatively superior tensile strength to that of all-cellulose composite films prepared by extracting cellulose and cellulose nano whisker (CNW) from corn straw using NaOH/thiourea as solvent [22].

During the comprehensive performance investigation of the prepared ACNC, it is also found to have a significant oxygen barrier effect, as shown in Figure 4f, revealing the influence of different contents of CNF on the oxygen permeability of the composite film material of cellulose. At RH50%, the oxygen permeability values of RC-C8 and RC-C12 were 86.94 and 63.83 cm^3^ μm/(m^2^ day atm), respectively. According to the reported criterion, a material with an oxygen permeability of 40–400 cm^3^·μm/(m^2^ day·atm) (RH50%, RT) is defined as high oxygen barrier [39]. In this case, RC-C8 and RC-C12 are both considered as high oxygen barriers. At a higher humidity of RH80%, RC-C8 and RC-C12 also exhibited good oxygen barrier capability, with oxygen permeability values of 690.37 and 430.54 cm^3^ μm/(m^2^ day atm), respectively. (At RH80%, RC-C0 film ruptured because of the pressure difference so the values cannot be obtained.) Morphological analysis shows that this may be associated with the uneven diffusion of solvent in the regeneration process, which leads to obvious defects in the cellulose membrane, and these are the main channels through which oxygen passes [40,41]. In our study, after the introduction of CNF, the oxygen resistance performance was reduced by more than 400 times. Oxygen barrier performance enhancement, on the one hand, results from strengthening the good compatibility with the substrate. On the other hand, CNF has a fibrous network distributed in the matrix, so oxygen molecules have to follow a “zigzag diffusion path” to permeate through the membrane. Thus, oxygen diffusion is suppressed with an increased diffusion length. Finally, when CNF content reached 12 wt%, the oxygen permeability of ACNC decreased to 63.83 cm^3^ μm/(m^2^ day atm). This is very competitive compared to common plastic films, such as Polystyrene (PS) (100–150 cm^3^ μm/(m^2^ day atm)), Poly(ethylene terephthalate)-12 (110 cm^3^ μm/(m^2^ day atm)), and Polypropylene-20 (1500–1800 cm^3^ μm/(m^2^ day atm)) [42]. High-performance thin films with low permeability are very attractive materials for electronic devices, such as flexible electrodes and organic light-emitting diodes (OLEDs). Excellent gas resistance is a basic requirement for packaging materials of electronic device encapsulation, because this type of film can effectively prevent the diffusion of oxygen into the conductive layer, increasing the life of electronic devices [43].

Compared to other all-cellulose nanocomposites/composites and cellulose-based composites in prior literature, the ACNC in this work has a relatively facile preparation at a moderate temperature, and also exhibit good mechanical and optical properties and oxygen barrier performance (Table S2). Compared to most commercial polyolefin films such as Polyethylene (PE) and Polypropylene (PP), the ACNC is biodegradable and has good mechanical properties. As shown in Figure S2, there is a ~70% weight loss of RC-C8 when being buried in the soil for 30 days, and these ACNC films all have higher tensile strengths over 63 MPa (notably, the tensile strength RC-C8 is as high as 87 MPa) than PE and PP, which show tensile strengths in a range of 20–40 MPa [44], making it a competitive green alternative to packaging bags, packaging tapes, etc. In addition, swelling properties are also important in terms of the application in packaging materials. Compared to literature data on water uptake for the all-cellulose nanocomposites of ~34% [8], RC-C8 has a lower water absorption of 21.3%, and these results are shown in Supplementary Materials. 

## 4. Conclusions

In this research, CNF-reinforced ACNC membranes were obtained and characterized. Compared with traditional preparation methods of all-cellulose composites, this study adopted a green and sustainable approach using a recyclable two-component IL/DMSO solvent system to subtly manipulate the distribution of CNF, which shows prospects for practical use. In terms of the crystallinity and morphology, the coexistence of cellulose I and cellulose II in the composite was confirmed by FTIR and XRD, and the crystallinity of the composite was improved. SEM images showed that ACNC exhibited a homogeneous morphology with appropriate roughness. Meanwhile, the mechanical properties and thermal stability of ACNC were also improved compared to a material based on regenerated cellulose without the addition of CNF. In addition, the obtained ACNC also had high optical transparency and good oxygen barrier performance. Based on these unique properties, bio-based ACNC has the potential to replace industrial polymer plastics. Taking all these results into account, among all ACNC samples with the added CNF contents of 2.00 wt%, 4.00 wt%, 8.00 wt%, and 12.00 wt%, RC-C8 had excellent thermal stability, high optical transmittance (86.5% at 550 nm), good mechanical properties (with tensile strength of 87 ± 4 MPa), outstanding oxygen barrier performance (86.94 cm^3^ μm/(m^2^ day atm) at RH50%), and other excellent functional properties (biodegradability and swelling properties), so it can be considered the most promising in various applications of encapsulation of electronic devices and other packaging materials.

## Data Availability

Data is contained within the article and supplementary materials.

## Supplementary Materials

The following supporting information can be downloaded at: https://www.mdpi.com/article/10.3390/membranes14010016/s1, Figure S1: Images of corn straw pulp dissolved in [Bu_4_N]^+^Ac^-^/DMSO at 60 °C at various time intervals, Figure S2: Weight loss of RC-C8 in the soil, Table S1: Weight of CNF in CNF/cellulose dispersion, Table S2: Comparison with other all-cellulose composites and cellulose-based composites. Table S3: Mechanical properties of the ACNC samples. References [27,45,46,47,48,49,50,51,52,53,54,55,56] are cited in the Supplementary Materials.
