# Supplementary material for: Green Preparation and Functional Properties of Reinforced All-Cellulose Membranes Made from Corn Straw
Source: Membranes (Basel). 2024 Jan 5;14(1):16. doi: 10.3390/membranes14010016 (PMC10821472; doi:10.3390/membranes14010016)
Supplement: Supplementary file 1 [file membranes-14-00016-s001.zip › membranes-2739022-supplementary.pdf]

Supplementary Materials

Green Preparation and Functional Properties of Reinforced All-Cellulose Membranes Made from Corn Straw

Wentao Zhang, Tianhao Wang, Zeming Jiang, Xin Gao, Changxia Sun and Liping Zhang *

College of Materials, Science and Technology, Beijing Forestry University, Beijing 100083, China; 18311395685@163.com (W.Z.); uyxdco@163.com (T.W.); ezxgsr@163.com (Z.J.); bozhnd@163.com (X.G.); fxctao@163.com (C.S.)

* Correspondence: zhanglp418@163.com

1. Preparation of ACNC

Table S1. Weight of CNF in CNF/cellulose dispersion.

Samples	CNF (g)
RC-C0	0
RC-C2	0.7
RC-C4	0.14
RC-C8	0.28
RC-C12	0.42

In cellulose solution, the $[Bu_4N]^+Ac^-$ and DMSO used were 13.02 g and 33.48 g. The DMSO used for CNF dispersion was 21.455 g.

2. Dissolution Processes of CP

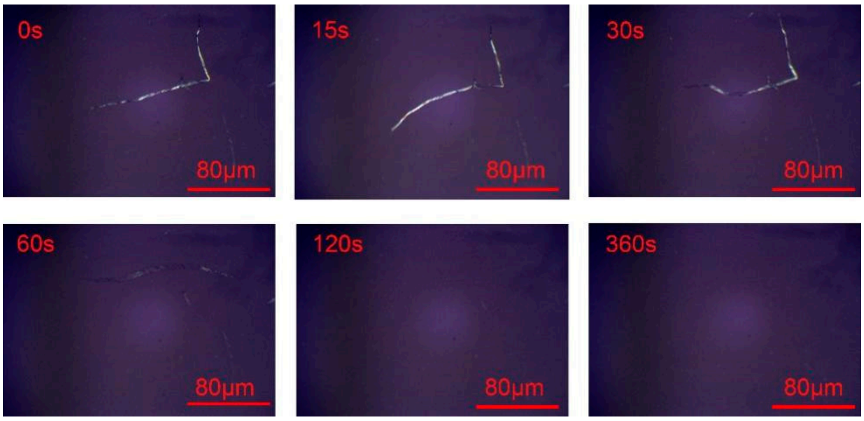

Figure S1. Images of corn straw pulp dissolved in $[Bu_4N]^+Ac^-$/DMSO at 60 °C at various time intervals: 0 s, 15 s, 30 s, 60 s, 120 s, 360 s.

3. Comparison of Preparation and Characteristics with Other Composites

Table S2. Comparison with other all-cellulose composites and cellulose-based composites.

Materials	Preparation					Characteristics			Ref.
	**Cellulose source**	**Method**	**Solvent System**	**Dissolution Condition**	**Film Processing**	**Tensile Strength (mpa)**	**Optical Transmittance**	**OP[a]**	
All-Cellulose Nano-composites/composites	Corn straw pulp (DP=1700-1750)	Hydrolysis and dissolution	$[B_4N]^+Ac^-$/DMSO	60 °C	Solution casting (Blade coating)	87	90.2% at 550 nm	63.83 cm^3 µm/(m^2 day atm) at RH50%	This work
	Corn stalk	TEMPO oxidation, acid–base method and a Soxhlet extraction	DMAc/LiCl	120 °C (With activation)	Solution casting (Blade coating)	52.0	N/A	N/A	[1]
	filter paper and rice husk	Soxhlet extraction and immersion	$[C_4mim]^+/Cl^-$	100 °C	Solution casting	57.5	N/A	N/A	[2]
	Filter paper	partial dissolution	[EMIM][OAc]	60 °C	Hot pressing	120	~80% at 550 nm	N/A	[3]
	Commercially obtained TEMPO-oxidized cellulose (made from wood bleached kraft pulp)	TEMPO oxidation	N/A[b]	N/A[b]	Vacuum filtration	167	~50% at 600 nm	1–2 mL µm m^{-2} day^{-1} kPa^{-1} at RH50%	[4]
regenerated cellulose/ZnO NP nanocomposite	Cotton origin	Activation and dissolution	DMAc/LiCl	(With activation)	Solution casting	116.18	79.05% at 660 nm	0.70 cc·µm m^{-2} day^{-1} kPa^{-1} at RH1%	[5]
Cellulose/zein composite	bleached kraft pulp of pine	hydrolysis and dissolution	NaOH/urea	cooling	Coagulation and hot pressing	49.54	N/A	2.16 cm^3·µm/(m^2·day·kPa)	[6]
Cellulose/graphene nanocomposite	Cotton linter (DP=640)	Activation and dissolution	DMAc/LiCl	100 °C (with activation)	Solution casting	148 MPa	67.6% (wavelength not provided)	N/A	[7]
Cellulose/GO nanocomposite	Organosolvent pulp from *Miscanthus x giganteus*	hydrolysis	acetone	60 °C	Drop coating	N/A	Below 80% at 500 nm	N/A	[8]
	N/A[b]	N/A[b]	$ZnCl_2/CaCl_2$/GO/water	65 °C	Solution casting	59.5	~75% at 550 nm	N/A	[9]

Cellu-lose/CNT nanocom-posite	Commercially obtained euca-lyptus dissolv-ing pulp (DP=652)	Dissolution, homogeniza-tion and cen-trifugation	NMMO/wa-ter	90 °C	Suction filtration and hot pressing	32	Below 25%	N/A	[10]
	Cotton linters pulp (10 × 10^4 g/mol)	Sonication, dissolution and centrifu-gation	NaOH/urea	−12 °C	Solution casting	60.6	Visually not transparent	N/A	[11]
ESO-plasti-cized EC	Commercially obtained EC	N/A[b]	acetone	N/A[b]	Solution casting	35.5	98% at 560 nm	10.0×10^{-10} cm^2/(s cmHg) at RH50%	[12]

[a] OP—oxygen permeability. Since thickness is not provided in some of the references, it is not possible to unify the units of OP. [b] The material is directly purchased from chemical reagent companies, so detailed information is not provided. TEMPO—2, 2, 6, 6-tetramethylpiperidine-1-oxyl-radical, DMAc—N, N-Dimethylacetamide anhydrous, $[C4mim]^+/Cl^-$—1-N-butyl-3-methylimidazolium chloride, GO—graphene oxide, CNT—carbon nanotube, NMMO—N-methyl -morpholine-N-oxide, ESO—Epoxidized soybean oil, EC—ethyl cellulose.

4. Mechanical Properties

Table S3. Mechanical properties of the ACNC samples.

Sample	Elongation-at-break (%)	Thickness (μm)	Tensile Strength (MPa)
RC-C0	10 ± 2	10 ± 1	54 ± 2
RC-C2	11 ± 2	8 ± 1	69 ± 1
RC-C4	13 ± 1	12 ± 2	73 ± 3
RC-C8	9 ± 2	9 ± 2	87 ± 4
RC-C12	8 ± 4	8 ± 3	63 ± 6

5. Biodegradability

5.1. Method

The biodegradability is evaluated based on the method reported in the prior literature [13]. The RC-C8 film was enclosed in a nylon mesh netting and buried about 10 cm beneath natural farmland soil. During 30 days of burying, every five days, the degraded RC-C8 film was taken out, rinsed with water, dried and weighed. The degradation time dependence of weight loss was obtained.

5.2. Results

The weight loss of RC-C8 in the soil is shown in Figure S2.

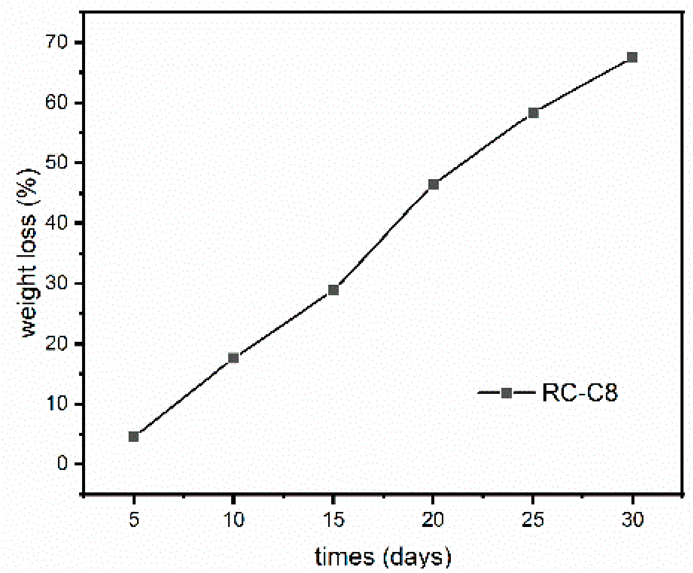

Figure S2. Weight loss of RC-C8 in the soil.

6. Swelling Properties

6.1. Method

To evaluate the water resistance of the obtained samples, the dried RC films were weighed (m_0) and immersed in deionized water in flasks, and taken out at regular intervals. The water that adhered to the film surface was absorbed with filter paper and the films were weighed again (m_t). The water absorption percent (W%) of the film was calculated (Equation (S1)). A total of three measurements were taken for each sample to guarantee data accuracy and repeatability.

$$W(\%) = (m_t - m_0)/m_0 \quad (1)$$

6.2. Results

The swelling test was used to indicate the water absorption, in order to further confirm the water stability of the composite films. After immersing the samples in water for over 24 h, the water absorption of the RC-C0 was ca. 86.3% upon reaching equilibrium. With the increasing content of cellulose, the water absorption of the films decreased from a value of 52.7% (RC-C2) to 21.3% (RC-C8). The water sorption in cellulose is mainly mediated by free hydroxyl groups in amorphous regions. The increase in the crystallization areas leads to a decrease in ACNC water absorption, thus resulting in a decrease in water absorption percent.

References

1. Bian, H.; Tu, P.; Chen, J.Y. Fabrication of all-cellulose nanocomposites from corn stalk. *J. Sci. Food Agric.* **2020**, *100*, 4390–4399, https://doi.org/10.1002/jsfa.10476.
2. Zhao, Q.; Yam, R.C.M.; Zhang, B.; Yang, Y.; Cheng, X.; Li, R.K.Y. Novel all-cellulose ecocomposites prepared in ionic liquids. *Cellulose* **2008**, *16*, 217–226, https://doi.org/10.1007/s10570-008-9251-3.
3. Chen, F.; Bouvard, J.-L.; Sawada, D.; Pradille, C.; Hummel, M.; Sixta, H.; Budtova, T. Exploring digital image correlation technique for the analysis of the tensile properties of all-cellulose composites. *Cellulose* **2021**, *28*, 4165–4178, https://doi.org/10.1007/s10570-021-03807-9.
4. Wakabayashi, M.; Fujisawa, S.; Saito, T.; Isogai, A. Nanocellulose Film Properties Tunable by Controlling Degree of Fibrillation of TEMPO-Oxidized Cellulose. *Front. Chem.* **2020**, *8*, 37, https://doi.org/10.3389/fchem.2020.00037.
5. Saedi, S.; Shokri, M.; Kim, J.T.; Shin, G.H. Semi-transparent regenerated cellulose/ZnONP nanocomposite film as a potential antimicrobial food packaging material. *J. Food Eng.* **2021**, *307*, https://doi.org/10.1016/j.jfoodeng.2021.110665.
6. Chu, Y.; Popovich, C.; Wang, Y. Heat sealable regenerated cellulose films enabled by zein coating for sustainable food packaging. *Compos. Part C: Open Access* **2023**, *12*, https://doi.org/10.1016/j.jcomc.2023.100390.
7. Zhang, X.; Liu, X.; Zheng, W.; Zhu, J. Regenerated cellulose/graphene nanocomposite films prepared in DMAC/LiCl solution. *Carbohydr. Polym.* **2011**, *88*, 26–30, https://doi.org/10.1016/j.carbpol.2011.11.054.

8. Nedilko, S.; Barbash, V.; Kleshonok, T.; Scherbatskii, V.; Shegeda, M.; Yashchenko, O.; Sheludko, V.; Gomenyuk, O. Morphology, Optical and Electronic Characteristics of Nanocellulose Filled with Microcrystalline Cellulose and Graphene Oxide. 2020 IEEE 10th International Conference Nanomaterials: Applications & Properties (NAP). Sumy, Ukraine: 09–13 November 2020; pp. 01NP06-1–01NP06-6.
9. Zhang, X.-F.; Song, L.; Wang, Z.; Wang, Y.; Wan, L.; Yao, J. Highly transparent graphene oxide/cellulose composite film bearing ultraviolet shielding property. *Int. J. Biol. Macromol.* **2019**, *145*, 663–667, https://doi.org/10.1016/j.ijbiomac.2019.12.241.
10. Cui, J.; Lu, P.; Li, Y.; Xu, K.; Li, Y.; Shen, H.; Liu, C.; Zhang, T.; Liu, D. The Flexible and Transparent Film Heaters Based on Regenerated Cellulose and Carbon Nanotubes. *Front. Energy Res.* **2022**, *10*, https://doi.org/10.3389/fenrg.2022.879257.
11. Xie, Y.; Xu, H.; He, X.; Hu, Y.; Zhu, E.; Gao, Y.; Liu, D.; Shi, Z.; Li, J.; Yang, Q.; et al. Flexible electronic skin sensor based on regenerated cellulose/carbon nanotube composite films. *Cellulose* **2020**, *27*, 10199–10211, https://doi.org/10.1007/s10570-020-03496-w.
12. Yang, D.; Peng, X.; Zhong, L.; Cao, X.; Chen, W.; Zhang, X.; Liu, S.; Sun, R. "Green" films from renewable resources: Properties of epoxidized soybean oil plasticized ethyl cellulose films. *Carbohydr. Polym.* **2014**, *103*, 198–206, https://doi.org/10.1016/j.carbpol.2013.12.043.
13. Tong, R.; Chen, G.; Tian, J.; He, M. Highly transparent, weakly hydrophilic and biodegradable cellulose film for flexible electroluminescent devices. *Carbohydr. Polym.* **2019**, *227*, 115366, https://doi.org/10.1016/j.carbpol.2019.115366.
